# Biomass-Derived-Carbon-Supported Spinel Cobalt Molybdate as High-Efficiency Electrocatalyst for Oxygen Evolution Reaction

**DOI:** 10.3390/molecules29204953

**Published:** 2024-10-19

**Authors:** Baoli Wang, Xiujiu Yang, Yan Chen, Jiahan Wang, Mingguo Lan, Kai Tang, Feng Yang

**Affiliations:** 1 Haikou Marine Geological Survey Center, China Geological Survey, Haikou 571127, China; 2 Technology Innovation Center for Analysis and Detection of the Elemental Speciation and Emerging Contaminants, China Geological Survey, Kunming 650111, China

**Keywords:** oxygen evolution reaction, CoMoO_4_ nanorod, electrocatalysis, water splitting

## Abstract

*Ananas comosus* leaves were converted to a porous graphitized carbon (GPLC) material via a high-temperature pyrolysis method by employing iron salt as a catalyst. A cobalt molybdate (CoMoO_4_)-and-GPLC composite (CoMoO_4_/GPLC) was then prepared by engineering CoMoO_4_ nanorods in situ, grown on GPLC. N_2_ adsorption–desorption isothermal curves and a pore size distribution curve verify that the proposed composite possesses a porous structure and a large specific surface area, which are favorable for charge and reactant transport and the rapid escape of O_2_ bubbles. Consequently, the as-synthesized CoMoO_4_/GPLC shows low overpotentials of 289 mV and 399 mV to afford the current densities of 10 mA cm^−2^ and 100 mA cm^−2^ towards the oxygen evolution reaction (OER), which is superior to many CoMoO_4_-based catalysts in previous studies. In addition, the decrease in current density is particularly small, with a reduction rate of 3.2% after a continuous OER procedure for 30 h, indicating its good stability. The excellent performance of the CoMoO_4_/GPLC composite proves that the GPLC carrier can obviously impel the catalytic activity of CoMoO_4_ by improving electrical conductivity, enhancing mass transport and exposing more active sites of the composite. This work provides an effective strategy for the efficient conversion of waste *ananas comosus* leaves to a biomass-derived-carbon-supported Co-Mo-based OER electrocatalyst with good performance, which may represent a potential approach to the development of new catalysts for OER, as well as the treatment of waste biomass.

## 1. Introduction

The rapid consumption and inadequate reserves of non-regenerated energy sources and their environmental consequences, originating from the emission of greenhouse gasses, impel the demand for the rational design and development of cost-effective methods to produce clean energy [1,2,3]. Hydrogen is considered to be a promising alternative to non-renewable energy because of its high energy density and pollution-free nature [4,5,6]. Owing to its high energy conversion efficiency, electrochemical water splitting through a cathodic hydrogen evolution reaction (HER) and an anodic oxygen evolution reaction (OER) is believed to be a potentially effective method to produce hydrogen [7,8,9,10]. However, the unsatisfactory efficiency of the inert reaction kinetics of OER is the dominating obstacle in these energy conversion and storage devices [11,12]. At present, the noble metal oxides of ruthenium (Ru) and iridium (Ir) exhibit the highest electrocatalytic performance towards OER, but their scarcity and high cost significantly hold back their extensive applications [13,14]. Therefore, it is imperative to develop abundant and low-cost catalysts to address these constraints.

Transition metal oxides (TMOs) seem to be good candidates for electrocatalytic OER because of their low cost, substantial deposits and good stability in the OER process [15]. Among many TMOs, manganese (Mn), iron (Fe), cobalt (Co) and nickel (Ni) are commonly used as active species for fabricating OER catalysts, in which various other transition metal elements can be employed as doping elements to develop metal oxides with specific structures and components, or to regulate the electronic properties of active sites. Therefore, binary TMOs, such as NiCo_2_O_4_ [16], CoMn_2_O_4_ [17], CoMoO_4_ [18,19,20], etc., have become a research hotspot due to their superior redox activity, large specific surface area and different crystal phase characteristics. However, pure CoMoO_4_ still shows undesirable OER performance because of its restricted active sites and poor electrical conductivity, which are both critical problems to be solved [21]. For instance, Zhang et al. [22] adopted the interface engineering approach to synthesize a P-CoMoO_4_/NiCoP nanowire–nanosheet composite on a nickel foam matrix, and achieved effective water dissociation and gas evolution in the process of water splitting. Using a simple and highly controllable two-step method, Wang et al. [23] designed a chrysanthemum-like electrocatalyst (Cu(OH)_2_@CoMoO_4_·0.9H_2_O/CF) which exhibited low overpotentials of 57 mV at 10 mA cm^−2^ towards an HER and 355 mV at 100 mA cm^−2^ towards an OER. When serving as a bifunctional electrocatalyst, it dissociated water at a cell voltage of 1.63 V to obtain a current density of 20 mA cm^−2^. Xie et al. [24] proposed fluorine-doped cobalt molybdate nanosheet arrays on graphite felt and efficiently stimulated OER kinetics with an overpotential of 256 mV at a current density of 10 mA cm^−2^, as well as a small Tafel slope of 64.4 mV dec^−1^ in an alkaline electrolyte. Luo et al. [25] reported excellent electrocatalytic performance of a CoMoO_4_-CoP heterostructure anchored on a hollow polyhedral N-doped carbon skeleton for water splitting applications. Evidently, it is reasonable to elaborately modulate the local electron using heterojunction fabrication, non-metal doping and morphological control to synergistically enhance the electrocatalyst’s OER performance.

In recent years, biomass-derived carbon materials have garnered a lot of research interest due to their inherent hierarchical porous structure, which provides a large available surface area and smooth transport channels for electrolyte ions. Additionally, most biomass contains N, S and P non-metal elements, or Fe, Se and K metal elements, so heteroatom self-doping can be achieved during the preparation procedure to generate accessional active sites in the as-synthesized carbon material, which makes them the most promising functional materials or catalyst carriers for energy storage and conversion. For instance, a N-doped few-layer porous carbon nanosheet catalyst was prepared by Liu et al. [26]. It was provided with more electrochemical active sites, a high specific surface area, a high atomic ratio of pyridinic/pyrrolic N and smaller mass transfer resistance, leading to good water splitting performance in 1 M KOH. Kumaresan et al. [27] synthesized *Acorus Calamus* plant-root-derived electrocatalysts with intrinsically implanted heteroatoms. The catalyst was found to have an excellent morphology, surface area, pore size and pore volume, and intrinsically doped N. When used as an electrocatalyst for HER and OER, it exhibited appreciable electrocatalytic activity, with overpotentials of 330 mV and 563 mV, respectively, vs. RHE at a current density of 10.0 mA cm^−2^. Additionally, an FeNi alloy and nitrogen-co-doped porous carbon (FeNi-NC) electrocatalyst was prepared by using peanut shells as precursors and a small amount of iron and nickel salts as non-precious metal sources [28]. The as-synthesized FeNi-NC catalyst exhibited not only excellent ORR electrocatalytic activities, but also satisfactory OER performance with similar activity to IrO_2_ in alkaline. Obviously, it is necessary to modify biomass-derived carbon using control strategies including heteroatom doping and defect introduction, as well as the loading of non-precious metals, to further enhance its catalytic activity [29,30].

**Scheme 1:**
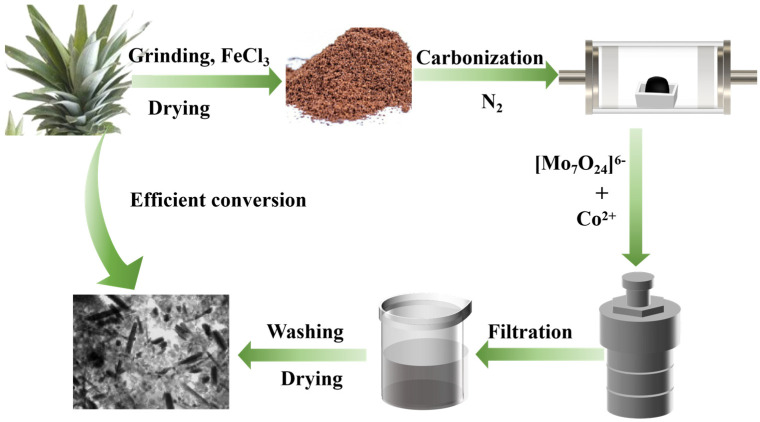
The preparation procedure of CoMoO_4_/GPLC.

*Ananas comosus* is native to tropical rainforests and tropical plateaus in Brazil and Paraguay. It mainly grows in the Hainan, Guangdong and Guangxi provinces in China, and *Ananas comosus* leaves are rich in cellulose, hemicellulose and lignin, which are the agricultural waste produced after harvesting *ananas comosus* fruit. Using the leaves as a sustainable resource can provide farmers with an additional source of income. This is particularly beneficial for the agricultural economies of many developing countries. On the basis of the above considerations, a facile and economical strategy was proposed to synthesize CoMoO_4_ nanorods supported on *Ananas comosus* leaf-derived graphitized carbon composite (CoMoO_4_/GPLC) using a hydrothermal method, with the preparation procedure shown in Scheme 1. The good dispersion of CoMoO_4_ and the small amount phosphorus and nitrogen doping increased the number of active sites and improved the hydrophilic behavior of the catalyst. Additionally, the synergistic effect between the CoMoO_4_ nanorods and GPLC nanoplate led to effective charge transfer and mass transport processes. Finally, the catalyst exhibited good OER performance and good long-term stability, superior to many reported CoMoO_4_-based oxide electrocatalysts.

## 2. Results and Discussion

### 2.1. Morphological and Structural Characterizations

**Figure 1:**
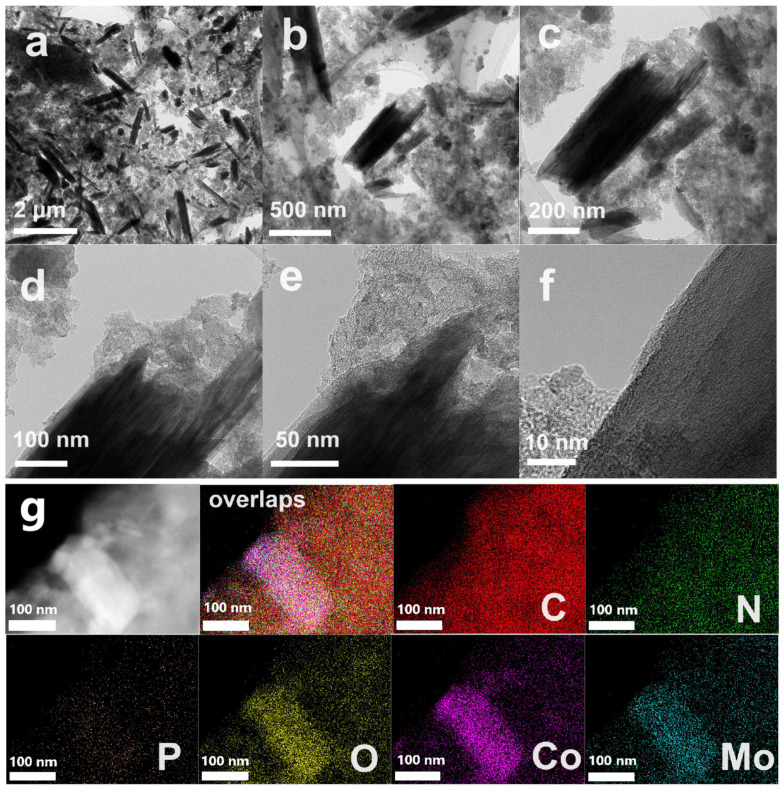
(**a**–**e**) TEM images of CoMoO_4_/GPLC with different magnifications, (**f**) HRTEM images and (**g**) EDS mappings of CoMoO_4_/GPLC.

The TEM images of the CoMoO_4_/GPLC composite from low to high magnifications show that a lot of nanorods dispersed on the surface of GPLC to form a unique architecture (Figure 1a–e). The measured sizes of the CoMoO_4_ nanorods range from 500 nm to 2 μm in length and 200 nm to 500 nm in diameter. An HRTEM image of the edge of a nanorod is displayed in Figure 1f, which verifies the effective crystallization of CoMoO_4_ nanorods. The interplanar distance of 0.278 nm for the (021) plane on the basis of the standard card of XRD (PDF# No. 21-0868) is in line with the XRD peak at 23.3° (The interplanar distance of 0.278 nm is ascribed to the (021) plane of CoMoO_4_ on the basis of the standard XRD card of PDF# No. 21-0868). This one-dimensional rod-like morphology is more conducive to mass and charge transport during electrochemical reactions [31]. To investigate the element distribution of CoMoO_4_/GPLC, an HAADF-STEM image and corresponding EDX maps were tested, with all the images depicted in Figure 1g. It is obvious that CoMoO_4_/GPLC is composed of C, N, P, O, Co and Mo elements, in which the reduced N and P is mainly due to the self-doping of GPLC. *Ananas comosus* requires a large amount of nitrogen and phosphate fertilizer to grow, resulting in the enrichment of N and P elements in *Ananas comosus* leaves, and further leading to the N and P self-doping of GPLC. All Mo, Co and O elements are uniformly distributed throughout the entire nanorod, demonstrating the formation of CoMoO_4_. The at.% values of C, N, O, P, Co and Mo are 54.91%, 5.18%, 25.97%, 4.10%, 3.84% and 6.00%, respectively. The doped N and P elements may provide more active sites for the electrochemical reaction.

**Figure 2:**
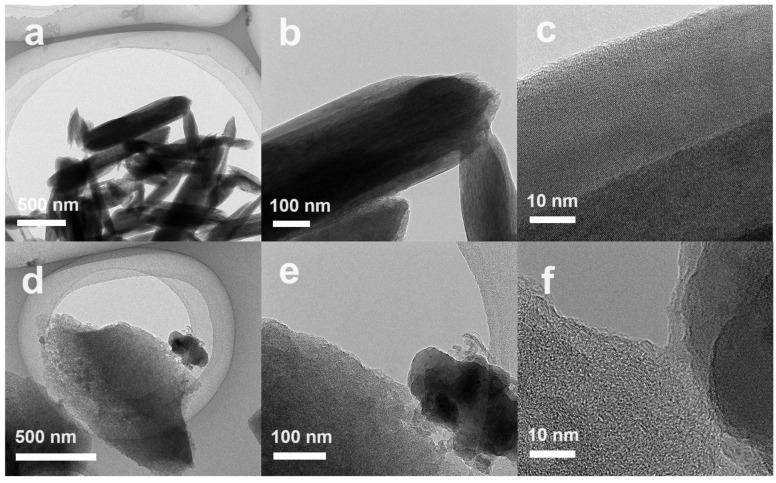
(**a**–**c**) TEM and HRTEM images of CoMoO_4_, (**d**,**e**) TEM and (**f**) HRTEM images of GPLC.

For the sake of investigating the effect of the carrier on the morphology and the structure of the material, TEM images of pure CoMoO_4_ and GPLC were also obtained, with the images displayed in Figure 2. It is found that CoMoO_4_ appears as nanorods with a length about 1 μm and a diameter of about 300 nm, which is similar to the dimensions of CoMoO_4_ in the CoMoO_4_/GPLC composite (Figure 2a,b). And the lattice fringe of the (021) crystal face of CoMoO_4_ can be observed in Figure 2c, verifying the formation of CoMoO_4_ with good crystallinity and orientation. The size and morphology of the CoMoO_4_ in CoMoO_4_/GPLC and the pure CoMoO_4_ vary slightly, which means that the carbon carrier does not bond with CoMoO_4_, and only loads CoMoO_4_ for good dispersion. The change in size and morphology may be due to the presence of GPLC in the hydrothermal solvent. It is clear that GPLC has a lamellar structure with different sizes, from several nanometers to several micrometers, which may be ascribed to the specific structure of the biomass (Figure 2c,d). Additionally, a crooked lattice fringe of graphite (002) can be observed, which indicates that the carbon skeleton has a certain degree of graphitization.

**Figure 3:**
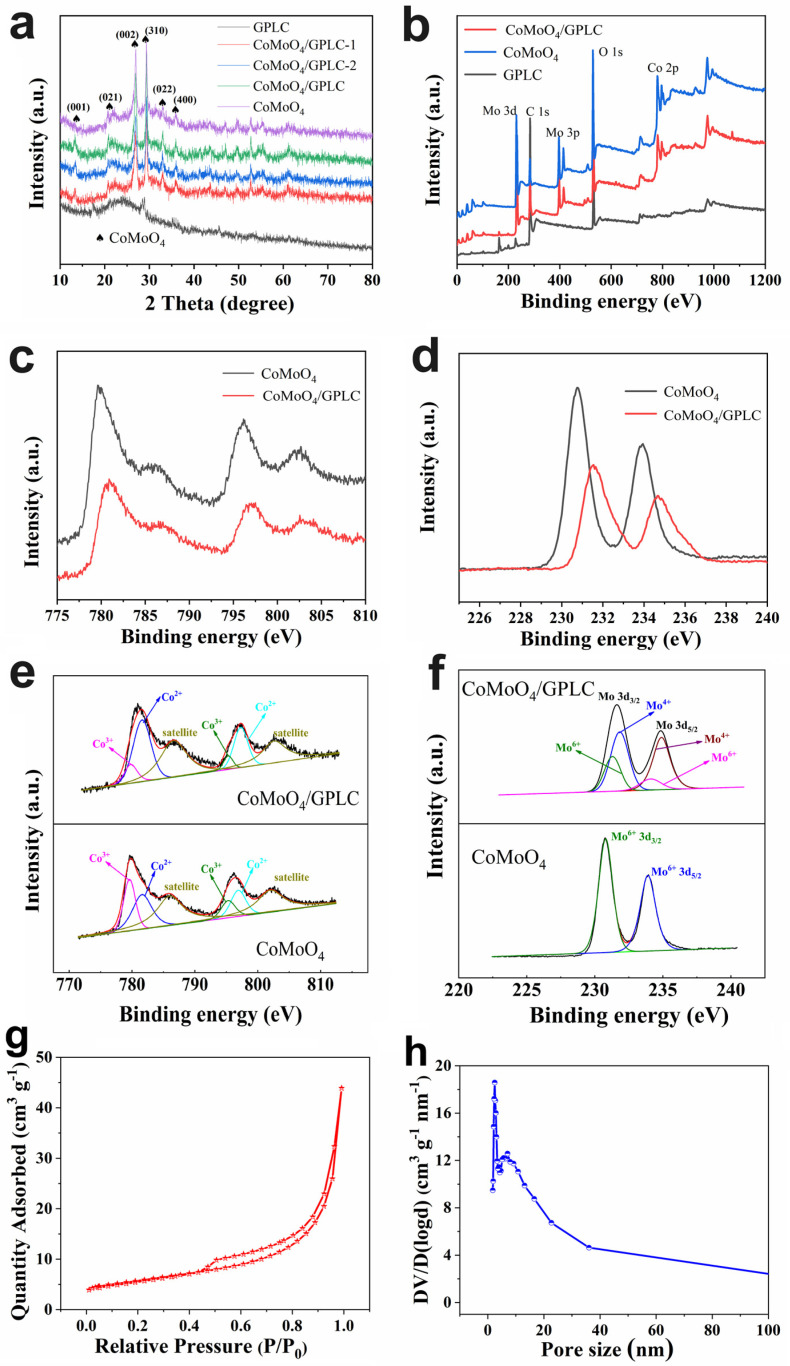
(**a**) XRD patterns of GPLC, CoMoO_4_, CoMoO_4_/GPLC−1, CoMoO_4_/GPLC and CoMoO_4_/GPLC−2; (**b**) full XPS spectra of GPLC, CoMoO_4_ and CoMoO_4_/GPLC; (**c**) fine Co 2p and (**d**) Mo 3d XPS spectra of CoMoO_4_ and CoMoO_4_/GPLC; high−resolution spectra of (**e**) Co 2p and (**f**) Mo 3d of CoMoO_4_ and CoMoO_4_/GPLC; (**g**) N_2_ adsorption−desorption isothermal curves and (**h**) pore size distribution curve of CoMoO_4_/GPLC.

The chemical composition and crystalline phase of the samples were investigated by XRD (Figure 3a). Concretely, in the XRD pattern of CoMoO_4_, the diffraction peaks located at 13.2°, 23.3°, 26.5°, 29.1°, 33.0° and 38.9° can be attributed to the (001), (021), (002), (310), (022) and (400) crystal planes of the CoMoO_4_ phase (PDF No. 21−0868), respectively. Additionally, the wide peak at about 25.2° in the pattern of GPLC is very consistent with the (002) plane of graphite. The XRD patterns of CoMoO_4_/GPLC-1, CoMoO_4_/GPLC and CoMoO_4_/GPLC−2 suggest the formation of CoMoO_4_ in the composite. However, the diffraction peaks of graphite cannot be distinctly observed in CoMoO_4_/GPLC−1, CoMoO_4_-GPLC and CoMoO_4_/GPLC−2, which is probably because the peak intensity of GPLC is weak or the peak overlaps with those of CoMoO_4_ in the range of 23° to 26°.

XPS measurements were carried out to further explore the chemical composition and the valence states of the samples, with the full XPS spectra depicted in Figure 3b, which proves that CoMoO_4_/GPLC mainly consists of C, Co, Mo and O elements. No significant peaks of N and P were found, probably because the amounts of N and P were relatively small or the peak intensities of the other elements were too strong, which causes covering of the N and P peaks. The Co 2p XPS spectra of CoMoO_4_ and CoMoO_4_/GPLC reveal similar peak positions, as shown in Figure 3c. However, the Mo 3d XPS spectrum (Figure 3d) of CoMoO_4_/GPLC shifts in the direction of high binding energy compared to that of pure CoMoO_4_, which reveals that the combination of GPLC and CoMoO_4_ affects the electronic structure of the Mo in the composite. In the fitting Co 2p spectra of CoMoO_4_ and CoMoO_4_/GPLC, the two strong peaks at 781.8 eV and 797.5 eV are ascribed to Co 2p_3/2_ and Co 2p_1/2_, which confirm the existence of Co^2+^ cations. A set of peaks at 779.6 eV and 794.8 eV signifies the existence of Co^3+^. And the other two peaks located at 785.9 eV and 802.4 eV are satellite peaks (Figure 3e). Evidently, the Co^3+^/Co^2+^ peak area ratio of CoMoO_4_/GPLC is significantly higher than that of CoMoO_4_. The Co 2p electron orbitals are active catalytic sites that accelerate the OER on account of the multiple valence states of Co [32]. The binding energies at 231.7 and 234.2 eV of CoMoO_4_ are split bimodal peaks of Mo^6+^ 3d_3/2_ and Mo^6+^ 3d_5/2_ (Figure 3f). As for CoMoO_4_/GPLC, the two peaks located at 232.8 eV and 235.4 eV owing to Mo^6+^ in the Mo 3d spectrum are successfully fitted with two more peaks at 232.20 eV and 234.7 eV assigned to Mo^4+^ and Mo^4+^, respectively. Additionally, it is found that the splitting width and energy difference between the Mo 3d_3/2_ and Mo 3d_5/2_ levels is approximately 3.1 eV, which is smaller with the previous literature report for Mo^6+^ [19]. The valences for Co and Mo in pure CoMoO_4_ must be Co^2+^ and Mo^6+^. The presence of Co^3+^ and Mo^4+^ is probably due to the doped elements of N and P species in the composite. It is possible that a small number of heteroatoms N and P may replace the lattice O in CoMoO_4_ during hydrothermal synthesis, resulting in a change in the valence state of the metal [33].

Different pore structures have a different functions in electrochemical performance. Thus, N_2_-adsorption–desorption isotherm curves were tested via the Brunauer–Emmett–Teller (BET) method to evaluate the porosity, specific surface area and pore size distribution of CoMoO_4_/GPLC. It can be seen that CoMoO_4_/GPLC shows typical characteristic Ⅳ adsorption isotherm curves and has H2 hysteresis loops in the relative pressure range of 0–1.0 P/P_0_, verifying the mesoporous structure of the sample (Figure 3g). The specific surface area is 163.5 m^2^ g^−1^. Based on the pore size distribution plot (Figure 3f), the pore size is mainly concentrated around 5 nm and 10 nm, and there are some larger pores in the range of 10 nm–100 nm. Pore sizes around 5 nm and 10 nm may be autogenous pores from GPLC, while larger pores may originate from the accumulations of CoMoO_4_. The large specific surface area can provide a lot of active sites for charge and mass adsorption, and the pore structure can offer a diffusion pathway for their migration, which may be favorable for in improving the electrocatalytic performance of the materials.

### 2.2. OER Activity

**Figure 4:**
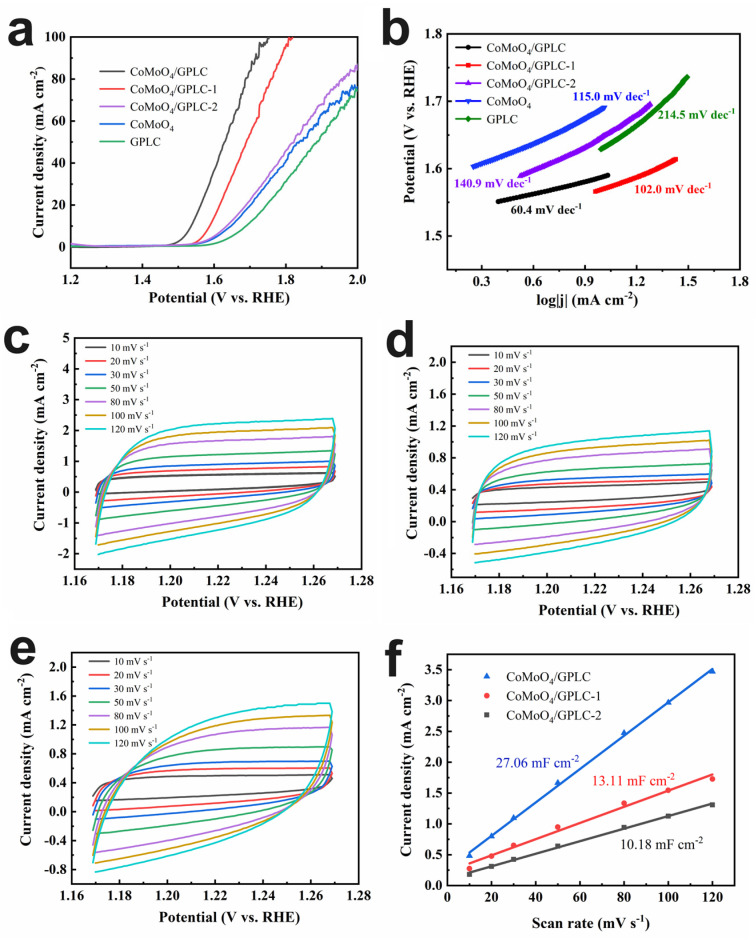
Electrocatalytic performance of various electrodes for OER in 1.0 M KOH. (**a**) LSV curves; (**b**) corresponding Tafel plots; CV curves of (**c**) CoMoO_4_/GPLC, (**d**) CoMoO_4_/GPLC−1 and (**e**) CoMoO_4_/GPLC−2; (**f**) plots of current density against scan rate.

An evaluation of the OER activity of the synthesized catalysts was performed by taking electrochemical measurements in 1.0 M KOH of electrolyte, with the LSV curves shown in Figure 4a. The overpotentials for CoMoO_4_/GPLC at current densities of 10 mA cm^−2^ and 50 mA cm^−2^ appear at 289 mV and 399 mV, respectively, which are observably smaller than those of CoMoO_4_/GPLC−1 (355 mV and 460 mV), CoMoO_4_/GPLC−2 (405 mV and 593 mV), CoMoO_4_ (417 mV and 608 mV) and GPLC (458 mV and 657 mV), indicating enhanced OER performance. This could be attributed to the even dispersion of CoMoO_4_ nanorods on the GPLC, more active sites originating from the trace P and N doping, and the appropriate load amount of CoMoO_4_ on the GPLC. Figure 4b shows Tafel plots to further study the OER kinetics of the as-synthesized catalysts. Generally, a smaller slope indicates faster reaction kinetics. CoMoO_4_/GPLC (60.4 mV dec^−1^) exhibits a smaller slope than those of CoMoO_4_/GPLC−1 (102.0 mV dec^−1^), CoMoO_4_/GPLC−2 (115.0 mV dec^−1^), CoMoO_4_ (140.9 mV dec^−1^) and GPLC (214.5 mV dec^−1^), confirming that CoMoO_4_/GPLC has the best OER kinetics. Since the values of the overpotential and Tafel slope allow a comparison of various electrocatalysts, and the Tafel slope value is often considered to be an activity metric, the overpotential and Tafel slope values of the reported CoMoO_4_-based electrocatalysts towards OER are listed in Table 1, which verifies that the performance of the proposed CoMoO_4_/GPLC is superior to most of the other catalysts.

To further determine the OER catalytic performance of the as-obtained catalysts, ECSA was evaluated by measuring the electrochemical double-layer capacitance (C_dl_) in the non-Faradaic potential range at various scan rates, with the CV curves shown in Figure 4c–e, which show that CoMoO_4_/GPLC occupies the biggest enveloped area. As depicted in Figure 4f, CoMoO_4_/GPLC possesses the biggest C_dl_ (27.06 mF cm^−2^), which is 2.1 and 2.7 times bigger than those of CoMoO_4_/GPLC−1 (13.11 mF cm^−2^) and CoMoO_4_/GPLC−2 (10.18 mF cm^−2^). This indicates that CoMoO_4_/GPLC has many more exposed electrochemically active sites towards OER. The reason that C_dl_ changes greatly with the variation in GPLC may be the synergistic effect caused by the appropriate CoMoO_4_ loading amount on the GPLC. When the loading amount increases, the adsorption channels and sites of the GPLC carriers may be covered, and the intrinsic active sites of CoMoO_4_ could also be reduced due to its aggregation. As the loading amount is reduced, the catalyst exhibits fewer intrinsic active sites, because CoMoO_4_ is the primary active species in the composite. Consequently, CoMoO_4_/GPLC achieves the maximum C_dl_ value. The corresponding ECSA value of CoMoO_4_/GPLC was calculated to be 676.5 cm^−2^ by dividing C_dl_ by the specific capacitance (C_s_) of an ideal planar (with an average value of 0.04 mF cm^−2^) [43]. Thus, these results demonstrate that CoMoO_4_/GPLC has a current density of 14.8 μA cm^−2^ at an overpotential of 289 mV, further confirming its outstanding intrinsic OER activity.

**Figure 5:**
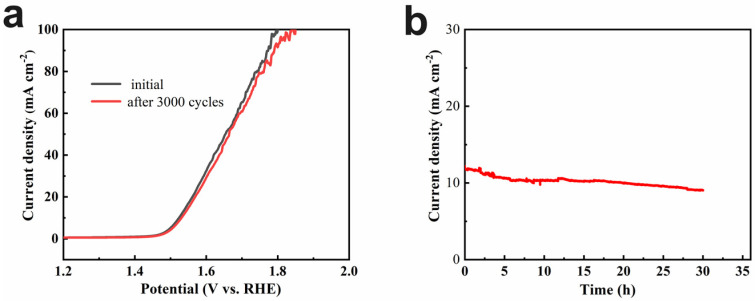
(**a**) LSV curves of CoMoO_4_/GPLC before and after 3000 cycles; (**b**) i−t curve of CoMoO_4_/GPLC for 30 h testing.

Taking into consideration the good catalytic performance of CoMoO_4_/GPLC towards OER, the electrochemical stability was further evaluated through the CV cycling and i-t methods. Sure enough, CoMoO_4_/GPLC exhibits excellent stability for OER in 1.0 M KOH. The LSV curves before and after 3000 CV cycles are almost constant, which indicates that CoMoO_4_/GPLC has good OER stability (Figure 5a). Moreover, after the consecutive OER operation for 30 h, the current density slightly changes, with a retention rate of 96.8%, further verifying CoMoO_4_/GPLC’s excellent long-term stability (Figure 5b). This phenomenon means that the stability of CoMoO_4_ can be obviously ameliorated by the carrier GPLC.

**Figure 6:**
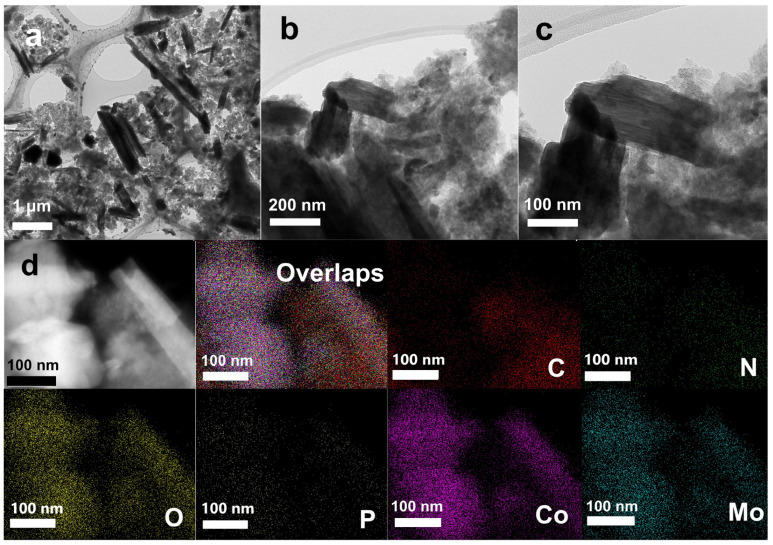
(**a**–**c**) TEM images of post-tested CoMoO_4_ with different magnifications; (**c**) TEM image and (**d**) the corresponding elemental mappings of post-tested CoMoO_4_ after 30 h stability assessed in 1 M KOH.

To investigate the morphological changes after the stability test, further TEM analyses were carried out, with the results depicted in Figure 6. It reveals a change in the morphology of the post-electrolysis sample compared to those of the fresh catalyst (Figure 1). In addition, the HRTEM images with the clear lattice fringe of the post-tested CoMoO_4_/GPLC cannot be gathered, which means that the degree of crystallinity is reduced after the durability test. Otherwise, it is possible that CoMoO_4_ is slowly converted to other species. Furthermore, the main elements Mo and Co are still evenly distributed with complete overlapping, as shown in Figure 6c.

The above results verify the good OER performance of the proposed CoMoO_4_/GPLC, which is comparable and even superior to previously reported CoMoO_4_-based electrocatalysts (Table 1). This good performance can be primarily attributed to the following factors: (i) the GPLC carrier effectively prevents the agglomeration of CoMoO_4_, which endows the catalyst with much more exposed intrinsic active sites; (ii) the nanorod structure of CoMoO_4_ and porous structure of the GPLC carrier accelerate charge transfer and mass transport; (iii) the co-doped N and P atoms in GPLC can be modulated and their electronic structure changed, resulting in more active sites, accelerated electron transfer and enhanced electrocatalytic performance; (iv) the combination of CoMoO_4_ with high intrinsic OER activity and GPLC with high conductivity shows a synergistic effect that leads to excellent OER efficiency.

## 3. Experimental Section

Information on the chemicals, apparatus, preparation method of CoMoO_4_/GPLC and electrochemical investigations is provided in the Supporting Information (SI).

## 4. Conclusions

In brief, a novel integrated binary hybrid composed of a CoMoO_4_/GPLC composite was prepared as an excellent electrocatalyst for OER. Owing to the synergistic effect between the CoMoO_4_ nanorods and GPLC nanoplates, CoMoO_4_/GPLC exhibits good OER activity with a low overpotential of 289 mV and achieves a current density of 10 mA cm^−2^, and the corresponding Tafel slope is only 60.4 mV dec^−1^, which is superior to many reported CoMoO_4_-based nanocatalysts. In addition, the catalyst has satisfactory long-term stability, maintaining 10 mA cm^−2^ for nearly 20 h. The reasons for its enhanced performance may be that the nitrogen and phosphorus doping of the carbon carrier provides more active sites, and the porous structure of the material provides more abundant migration channels for the reactants and products. Therefore, the combination of different nanomaterials with designed structures is a promising strategy to enhance catalytic performance, which offers an effective path for the synthesis of non-noble-metal-based catalysts.

## Figures and Tables

**Table 1:** Comparison of the electrochemical parameters of different CoMoO_4_-based electrocatalysts towards OER in alkaline electrolyte.

Catalyst	η (mV vs. RHE)	Tafel Slope (mV dec^−1^)	Electrolyte (KOH)	Refs.
CoMoO_4_/GPLC	η_10_, 289	60.4	1.0 M	This work
CoMoO_4_ flowers	η_10_, 312	56	1.0 M	[34]
NF/H-CoMoO_4_	η_10_, 295	/	1.0 M	[35]
Fe_0_._5_Co_0_._5_MoO_4−x_S_x_	η_10_, 268	87	1.0 M	[36]
CoMoO_4_ nanorods	η_10_, 343	67	1.0 M	[37]
CoMoO_4_-NiMoO_4_	η_10_, 300	68	1.0 M	[38]
CoMoO_4_/G	η_10_, 327	43	1.0 M	[39]
CoMoO_4_ nanotube	η_10_, 315	108	1.0 M	[40]
rGO/CoMoO_4_	η_10_, 475	167	1.0 M	[41]
NiCo_2_O_4_@CoMoO_4_/NF−7	η_20_, 265	102	1.0 M	[42]

## Data Availability

Data are contained within the article.

## Supplementary Materials

The following supporting information can be downloaded at: https://www.mdpi.com/article/10.3390/molecules29204953/s1.
