# Supplementary material for: Biomass-Derived-Carbon-Supported Spinel Cobalt Molybdate as High-Efficiency Electrocatalyst for Oxygen Evolution Reaction
Source: Molecules. 2024 Oct 19;29(20):4953. doi: 10.3390/molecules29204953 (PMC11510036; doi:10.3390/molecules29204953)
Supplement: Supplementary file 1 [file molecules-29-04953-s001.zip › molecules-3206143-supplementary.pdf]

Biomass-Derived-Carbon-Supported Spinel Cobalt Molybdate as High-Efficiency Electrocatalyst for Oxygen Evolution Reaction

Baoli Wang[a1], Xiujiu Yang[a1], Yan Chen[a], Jiahan Wang[a], Mingguo Lan[b], Kai Tang[a]*, Feng Yang[a]*

[a] Haikou Marine Geological Survey Center, China Geological Survey, Haikou, 571127, China

[b] Technology Innovation Center for Analysis and Detection of the Elemental Speciation and Emerging Contaminants, China Geological Survey, Kunming, 650111, China

[*] Corresponding authors.

E-mail address: likeyangff@126.com (F. Yang); 18976530623@163.com (K. Tang)

[1.] Baoli Wang and Xiujiu Yang contributed equally to this paper.

3.1. Chemicals

$Co(NO_3)_2·6H_2O$ and $(NH_4)_6Mo_7O_{24}·4H_2O$ were purchased from Shanghai Aladdin Biochemical Technology Co. Ltd. (China) and used as received. The ananas comosus leaves were collected from the local ananas comosus plantations. 5 wt % Nafion ethanol solution (Beijing Honghaitian Tech. Co. Ltd., China) are commercially sourced and used without further purification. All solutions applied in the synthesis process and electrochemical experiments were prepared with ultrapure water, which was filtered by Milli−Q IQ 7000 (Merck Millipore, Germany).

3.2 Apparatus

Electrochemical measurements were carried out on CHI 660E electrochemical workstation (Shanghai Chenhua Instrument Co. Ltd., China) with a conventional three−electrode system, including glassy carbon electrode (GCE, Ø = 3 mm) as

working electrode, carbon rod electrode (Ø = 3 mm) and saturated calomel electrode (SCE) as the counter electrode and the reference electrode. Transmission electron microscopy (TEM) was performed using a JEM−2010F microscopy (JEOL, Japan) operated at 200 kV. X−ray photoelectron spectroscopy (XPS) was carried out on an AXIS HIS 165 spectrophotometer (Kratos Analytical, UK). X−ray diffraction (XRD) experiments were tested on a D/Max−2500 V X−ray diffractometer (Rigaku, Japan) with Cu−Kα_1 radiation. N_2 adsorption−desorption isotherms were carried out using surface area and porosity analyzer (ASAP 2020, Micromeritics). Raman spectra of the samples were recorded on a LabRAM HR Evolution (Horiba Scientific, France) at a 514 nm excitation wavelength.

3.3 Preparation of **$CoMoO_4$/GPLC**

Firstly, 10.0 g *ananas comosus* leaves were cut into small pieces and dried at 80 °C in an oven followed by ground into powder and soaking in 500 ml 0.1 mol/L $FeCl_3$ solution for 24 h, and then filtered and dried again at 80 °C to gain a dried brown powder. Secondly, the powder was carbonized at 900 °C for 3 h under N_2 airflow with a heating gradient of 5 °C min^{-1} and soaked with concentrated HCl for 2 h followed by washing with ultrapure water to get a black product (denoted as GPLC). Thirdly, 0.275 g $(NH_4)_6Mo_7O_{24}·4H_2O$, 0.16 g $Co(NO_3)_2·6H_2O$ and 0.13 g GPLC were dissolved in 20 mL ultrapure water in a polytetrafluoroethylene reactor with magnetic stirring to form a homogeneously suspension, and transferred into a hydrothermal reactor followed by maintaining at 130 °C for 24 h. Finally, the obtained solid was filtered, washed with ultrapure water and absolute ethanol for several times, and dried at 80 °C for 12 h to get the final product (named as $CoMoO_4$/GPLC). The control sample $CoMoO_4$/GPLC−1 was prepared by reducing the amounts of GPLC by half and $CoMoO_4$/GPLC−2 was prepared by doubling the amounts of GPLC. For comparison, the pure $CoMoO_4$ were synthesized under similar conditions except for the addition of GPLC. The synthesis flow chart is shown in Scheme 1.

3.4. Electrochemical investigations

For preparing the working electrodes, 3.0 mg of the prepared samples were dispersed into 1.0 mL of the mixture of ethanol and ultrapure water (volume

ratio=1:1), followed by adding 30 μL 5.0 wt % Nafion ethanol solution and ultrasonic treatment for 30 min to get the uniform catalysts suspension. Then 6 μL suspension was dipped casted onto the polished GCE surface and dried under the ambient temperature for 2 h to obtain the working electrodes. The area of the used GCE is 0.07065 cm^2. The final loading was about 0.247 mg cm^{-2} for all the as−prepared catalysts.

Electrochemical measurements were carried out with all potentials reference to the reversible hydrogen electrode (RHE) based on the following formula: $E_{RHE} = E_{SCE}+0.059\times pH + E^0_{SCE}$ ($E^0 = 0.242$ V). Overpotential (η) was calculated according to the equation as: $\eta = E_{RHE} - 1.23$ V.

Linear sweep voltammetry (LSV) was operated in 1.0 M KOH solution with a scan rate of 5 mV s^{-1} after cyclic voltammetry (CV) was performed within 100 cycles to stabilize the current density. The long−term stability was evaluated by the amperometric i−t method. The electrochemical active surface area (ECSA) was assessed by electrochemical double layer capacitance (C_{dl}) and examined by CV, which was performed at different scan rates (from 10 mV s^{-1} to 120 mV s^{-1}) in the non−Faradaic current region (0.1−0.2 V vs. SCE).
